# Elevated level of serum human epididymis protein 4 (HE4) predicts disease severity and mortality in COVID-19 pneumonia

**DOI:** 10.1186/s12890-023-02811-y

**Published:** 2023-12-16

**Authors:** Renáta Sütő, Marianna Pócsi, Zsolt Szabó, Zsolt Fejes, Gergely Ivády, György Kerekes, Miklós Fagyas, Attila Nagy, Zoltán Szentkereszty, János Kappelmayer, Béla Nagy

**Affiliations:** 1 Department of Laboratory Medicine, Faculty of Medicine, University of Debrecen, Debrecen, Hungary; 2 Doctoral School of Kálmán Laki, Faculty of Medicine, University of Debrecen, Debrecen, Hungary; 3 Gyula Kenézy Campus, Intensive Care Unit, Faculty of Medicine, University of Debrecen, Debrecen, Hungary; 4 Department of Internal Medicine, Intensive Care Unit, Faculty of Medicine, University of Debrecen, Debrecen, Hungary; 5 Division of Clinical Physiology, Department of Cardiology, Faculty of Medicine, University of Debrecen, Debrecen, Hungary; 6 Department of Preventive Medicine, Faculty of Public Health, University of Debrecen, Debrecen, Hungary

**Keywords:** SARS-CoV-2, Inflammation, COVID-19 Disease, Pneumonia, Biomarker, HE4, Outcome

## Abstract

**Background:**

We retrospectively analyzed serum level of human epididymis protein 4 (HE4) as a pulmonary inflammatory biomarker in patients with COVID-19 pneumonia in association with disease severity and outcome.

**Methods:**

Ninety-nine (40 critically ill, 40 severe and 19 mild) COVID-19 patients and as controls 25 age- and sex-matched non-COVID-19 bacterial sepsis subjects were included. Serum HE4 was measured by an immunoassay (Architect^®^ i1000SR, Abbott) in the baseline samples of all study participants obtained at intensive care unit (ICU) admission or during outpatient clinic visit and follow-up sera were available in case of 30 COVID-19 subjects with life-threating conditions. Associations were studied between serum HE4, routinely available laboratory parameters, clinical characteristics, and disease progression.

**Results:**

Baseline HE4 level was significantly higher (*P* < 0.0001) in critically ill (524.7 [300.1–1153.0] pmol/L) than severe COVID-19 subjects (157.4 [85.2-336.9] pmol/L) and in mild SARS-CoV-2 infection (46.7 [39.1–57.2] pmol/L). Similarly increased HE4 concentrations were found in bacterial sepsis (1118.0 [418.3–1953.0] pmol/L, *P* = 0.056) compared to critically ill COVID-19 individuals. Serum HE4 levels significantly correlated with age, SOFA-score, inflammation-dependent biomarkers, and the degree of lung manifestation evaluated by chest CT examination in ICU COVID-19 individuals. Based on ROC-AUC curve analysis, baseline HE4 independently indicated the severity of COVID-19 with an AUC value of 0.816 (95% CI [0.723–0.908]; *P* < 0.0001), while binary logistic regression test found HE4 as an independent prognostic parameter for death (OR: 10.618 [2.331–48.354]; *P* = 0.002). Furthermore, COVID-19 non-survivors showed much higher baseline HE4 levels without a substantial change under treatment vs. survivors (*P* < 0.0001). Finally, pre-treatment HE4 level of ≥ 331.7 pmol/L effectively predicted a larger risk for mortality (Log-Rank *P* < 0.0001) due to severe COVID-19 pneumonia.

**Conclusion:**

Elevated serum HE4 level at ICU admission highly correlates with COVID-19 severity and predicts disease outcome.

## Background

Since the outbreak of the Coronavirus disease 2019 (COVID-19) pandemic caused by the severe acute respiratory syndrome coronavirus 2 (SARS-CoV-2) beta coronavirus, more than 767.5 million confirmed SARS-CoV-2 positive cases and over 6.9 million COVID-19-related deaths have been reported worldwide according to the World Health Organization (WHO, https://covid19.who.int, as of 28 June 2023). Most infected individuals had no or only mild symptoms, however, 4.3% of patients required admission to the intensive care unit (ICU) and died because of severe pneumonia and multiorgan failures [1].

Effective clinical biomarkers for monitoring of COVID-19 disease are essential to prevent severe consequences or death that is why validation of blood-based biomarkers in COVID-19 is still necessary for daily routine [2]. For instance, complement overactivation and consumption [3], plasma tissue plasminogen activator (tPA) and plasminogen activator inhibitor-1 (PAI-1) [4] and ACE2 activity [5] have recently been tested as new predictors of COVID-19 severity and outcome. However, such retrospective investigations are still being performed to discover additional parameters which would be clinically useful in case of a future COVID-19 pandemic [2].

Human epididymis protein 4 (HE4) is a small secretory glycoprotein, which was first described with a suspected role in the maturation of the sperms over 30 years ago [6]. Since then, HE4 was reported to be expressed in other human organs, such as salivary glands, the epithelial cells of the oral and nasal cavity as well as in the airways [7]. HE4 is the product of the *WFDC2* gene and is a member of the whey acidic protein (WAP) family that is homologous to other serine proteinase inhibitors, comprising elafin and secretory leukocyte protease inhibitors (SLPIs) [8]. Similar to these family proteins, HE4 also displays a variety of functions in the lung as an anti-proteinase in the frame of epithelial host defenses [9]. In parallel, this overexpressed protein has been widely utilized in tumor profiling, e.g., in epithelial ovarian cancer [10] and lung tumor [11].

The lung is the main affected organ in COVID-19 disease, and lung damage accompanied with exudative diffuse alveolar damage with hyaline membrane formation, pneumocyte type 2 hyperplasia and a massive secretion of pro-inflammatory cytokines is the leading cause of death in most patients [12]. Recently, serum HE4 concentration was reported to be markedly increased in sepsis-associated acute respiratory distress syndrome (ARDS) that predicted poor prognosis [13, 14]. In relation to COVID-19, only two publications are available in terms of the use of serum HE4 as a disease severity biomarker [15, 16]. However, other clinical aspects have not been clarified in these studies for the application of this protein in very severe SARS-CoV-2 infection caused pneumonia.

In this study, our aims were (i) to determine baseline serum HE4 levels to compare these values in critically ill, severe and mild COVID-19 patients, (ii) to analyze the direct relationship between serum HE4 before treatment and the progression of the disease using various statistical tests, (iii) to correlate HE4 level with routinely measured prognostic markers in COVID-19, such as ferritin and total lactate dehydrogenase (LDH) activity as well as the degree of lung manifestation evaluated by chest CT, and (iv) to predict the outcome of the disease using baseline HE4 results in COVID-19 subjects treated at ICU.

## Methods

### COVID-19 and bacterial sepsis patient cohorts

In this retrospective clinical study, 99 COVID-19 patients at the age of between 39 and 87 years were involved at the Clinical Center and Gyula Kenézy Campus, University of Debrecen, Debrecen, Hungary. At admission, these individuals suffered from (i) life-threatening ARDS, or (ii) severe, but clinically stable pneumonia, or (iii) only mild respiratory symptoms. All subjects were confirmed to be positive for SARS-CoV-2 by reverse transcription polymerase chain reaction (RT-PCR) test of a nasopharyngeal swab. Critically ill and severe patients were then transferred to the ICU at either center, while mild COVID-19 subjects did not need any hospitalization. Study participants were divided into three subgroups according to the severity of their clinical state: 40 subjects were categorized as “critically ill COVID-19”, 40 patients were characterized as “severe COVID-19”, while 19 individuals with non-severe disease were subgrouped into “mild COVID-19” (Table 1). None of these COVID-19 subjects suffered from any bacterial infection at the time of blood sampling. All ICU patients received the following standard treatment: oral or intravenous dexamethasone and antiviral therapy, remdesivir and erythromycin, thrombosis and gastric ulcer prophylaxis, vitamin C and D supplementation and O_2_ support or invasive ventilation corresponding to the severity of the respiratory failure. In the critically ill cohort, continuous renal replacement therapy was also applied in 54% of these patients due to acute kidney dysfunction. The exclusion criteria included cancer, autoimmune disease, pregnancy, and chronic lung disease. Disease severity was defined based on the Sequential Organ Failure Assessment (SOFA) scores and Horowitz index (P/F) determined by the clinicians. In addition, pre-existing comorbidities, COVID-19 specific treatment, the degree of pulmonary involvement based on chest CT, administration of invasive ventilation, the length of hospital stay, and the clinical outcome were recorded at the ICU wards. To assess the functional lung involvement and the impairment of organ oxygenation: the PaO_2_/FiO_2_ (Partial arterial pressure of oxygen (PaO_2_, mmHg) / Fraction of inspired oxygen (FiO_2_)  (P/F) index [17]), also known as Horowitz index was calculated by the clinicians for all patients with COVID-19. An index below 100 mmHg was defined severe, 100–200 mmHg moderate, and 200–300 mmHg mild ARDS. Parallelly, 25 critically ill bacterial sepsis individuals with negative COVID-19 RT-PCR test result were treated at the Department of Internal Medicine, ICU, University of Debrecen and participated in this study as a control group. The subjects of this control group were age- and sex-matched to both COVID-19 cohorts and had SOFA scores of (median, IQR) 9 (5–10) (Table 1). Sepsis was diagnosed based on the American College of Chest Physicians/Society of Critical Care Medicine Consensus criteria [18]. Two-thirds of these patients had a positive hemoculture (*e.g., Escherichia coli*, *Klebsiella pneumoniae*, *Enterococcus faecalis*, or *Streptococcus pneumoniae*, etc.), while the rest of individuals were culture-negative.

**Table 1:** Baseline demographical, clinical, and routine laboratory characteristics of 99 COVID-19 patients divided into three subgroups based on severity of the disease and 25 severe non-COVID-19 bacterial sepsis subjects. Data are expressed as median with IQR. For statistical analyses, Mann-Whitney U test or Fisher’s exact test was used, as appropriate. Significant differences were found in different comparisons indicated with variable symbols as follows: ^***^p < 0.0001, ^**^p < 0.001, ^*^p < 0.05 in case of comparison between critically ill vs. severe COVID-19 patients; ^###^*p* < 0.0001, ^##^*p* < 0.001, ^#^*P* < 0.05 when comparison between critically ill COVID-19 vs. critically ill bacterial sepsis patients, ^‡‡‡^*p* < 0.0001, ^‡‡^*p* < 0.001, ^‡^*P* < 0.05 when comparison between severe vs. mild COVID-19 patients. Abbreviations: SOFA-score: Sequential Organ Failure Assessment score, CRP: C-reactive protein, PCT: procalcitonin, IL-6: interleukin-6, GFR-CKD-EPI: Glomerular filtration rate - Chronic Kidney Disease Epidemiology Collaboration, AST: aspartate transaminase, ALT: alanine aminotransferase, LDH: lactate dehydrogenase, WBC: white blood cell, PLT: platelet, MPV: mean platelet volume, ACE2: Angiotensin-converting enzyme 2, n.d.: not determined

Variables	Critically ill COVID-19 (*n* = 40)	Severe COVID-19 (*n* = 40)	Mild COVID-19 (*n* = 19)	Critically ill bacterial sepsis (*n* = 25)
**Age (years) (median, IQR)**	65 (55–75)	61 (52–66)	63 (56–72)	68 (49–74)
**Sex (M/F)**	23/17	26/14	7/12	14/11
**Hospital stay (days) (median, IQR)**	9.5 (6–18)	10 (6–13)	0	10 (7-12.5)
**Mechanical ventilation (y/n, %)**	37/3 (93)^***^	2/38 (5)	-	11/14 (44)^#^
**SOFA-score**	12 (10–14)^***^	7 (3–10)	-	9 (5–10)
**Horowitz index (P/F) (median, IQR)**	95 (65–139)^**^	154 (100–277)	-	n.d.
**Lung manifestation by CT (%) (median, IQR)**	70 (55–80)^*^	55 (25–70)	-	n.d.
**Mortality (n, %)**	36 (90)^***^	3 (7.5)	0 (0)	11 (44)^#^
**CRP (mg/L)**	180 (131–271)^**^	112 (30–241)	2.3 (0.9–12.8)^‡‡‡^	175 (142–263)
**PCT (µg/L)**	0.5 (0.3–2.9)^***^	0.05 (0.05–0.21)	n.d.	9.0 (4.5–26.5)^###^
**IL-6 (ng/L)**	96.5 (38.8–330)^***^	38.7 (3.8–52.0)	n.d.	162 (61.9-835.3)
**Ferritin (µg/L)**	1271 (776–2455)^***^	613 (345–1031)	167 (30–468)^‡‡^	n.d.
**Urea (mmol/L)**	8.6 (6.2–13.8)^***^	5.5 (4.3–6.7)	5.3 (4.9-6.0)	12.7 (10.1–19.8)^##^
**Creatinine (µmol/L)**	109.0 (82.5-159.8)^**^	87.0 (79.3–98.5)	66.0 (59.0–78.0)^‡^	219 (93.5-313.5)^#^
**GFR CKD-EPI (mL/min/1.73 m2)**	59.5 (36.2–71.5)^*^	67.0 (60.0-77.8)	88.0 (73.2–90.0)^‡^	22.0 (15–58)^#^
**AST (U/L)**	49.0 (38.5–55.8)^*^	36.5 (27.3–50.3)	19.5 (15.0-25.3)^‡^	45.0 (22.5–181)
**ALT (U/L)**	36.5 (23.0–69.0)	36.0 (26.3–54.0)	22.0 (14.5–29.8)^‡^	42.0 (13.5-193.5)
**LDH (U/L)**	781 (674–1083)^***^	530 (393–748)	192 (179–244)^‡‡^	326 (251–455)^###^
**WBC count (G/L)**	9.1 (6.6–13.8)^*^	6.7 (5.4–10.1)	7.8 (5.9–9.5)	13.9 (7.7–19.8)^#^
**Hemoglobin (g/L)**	131 (119–147)	140 (127–151)	136 (131–150)	108 (93–127)^###^
**PLT count (G/L)**	237 (162–293)	213 (149–269)	239 (177–307)	174 (61–224)^#^
**MPV (fL)**	8.3 (7.6–9.9)	8.1 (7.2–8.8)	9.2 (7.8–10.8)	11.7 (10.5–12.3)^###^
**ACE2 activity (mU/L)**	50.5 (32.6–80.4)^**^	34.7 (25.4–49.7)	n.d.	n.d.
**Hypertension (y/n, %)**	37/3 (93)^*^	28/12 (70)	13/6 (68)	19/6 (76)^##^
**Diabetes mellitus**	10/30 (25)	12/28 (30)	3/16 (16)	12/13 (48)
**Cardiac decompensation**	14/26 (35)	8/32 (20)	0/0 (0)	5/20 (20)
**Atrial fibrillation**	12/28 (30)^*^	4/36 (10)	0/0 (0)	7/18 (28)
**Renal insufficiency**	13/27 (32.5)^**^	3/37 (7.5)	0/0 (0)	14/11 (56)

### Laboratory analyses

All study individuals had baseline peripheral venous blood samples drawn within the first 24 h of ICU admission or during outpatient clinic appointment prior to any SARS-CoV-2 infection or sepsis related treatment, and follow-up samples were available before death or discharge of patients in case of 30 COVID-19 patients. Routinely available laboratory serum tests were performed at the Department of Laboratory Medicine, University of Debrecen. Sera were stored at -70 °C; the analysis of serum HE4 was retrospectively performed by an automated immunoassay for HE4 (Architect^®^ i1000SR, Abbott Diagnostics, Wiesbaden, Germany). In parallel, the analysis of serum ACE2 activity was performed by a specific quenched fluorescent substrate (obtained from http://peptide2.com) as reported earlier [19]. Routinely available laboratory serum tests, i.e., C-reactive-protein (CRP), procalcitonin (PCT), interleukin-6 (IL-6), and ferritin were determined by electro-chemiluminescent immunoassays on a Cobas^®^ e411 analyzer (Roche Diagnostics, Mannheim, Germany), while enzyme activities (i.e., AST, ALT, LDH) and creatinine with urea levels were analyzed by kinetic colorimetric assays on a Cobas^®^ 8000 instrument (Roche Diagnostics). In parallel, hematology parameters, i.e., white blood cell (WBC) count, and platelet (PLT) count, hemoglobin concentration and mean platelet volume (MPV) were determined by an Advia^®^ 2120 Hematology System analyzer (Bayer Diagnostics, Tarrytown, NJ, USA).

### Statistical analyses

Before the enrollment of these study participants, a prior sample size calculation was performed using the Intercooled Stata v17 with a power level of 90% and an α level of 0.05 using means (145.7 and 284.0 pmol/L) and standard deviations (118.4 and 201.6 pmol/L) of serum HE4 measured in severe and critically ill COVID-19 patient cohorts based on a previous study [16]. Accordingly, the estimated required sample sizes were 30 per study subgroup. Kolmogorov–Smirnov test was used for evaluation of the normality of data. Results are expressed as median with interquartile range (IQR). To compare the data of two groups, we applied Mann–Whitney U test or Fisher’s exact test, as appropriate. Comparison of multiple groups was performed by Kruskal-Wallis test with Dunn’s multiple comparisons test. Serum HE4 values in baseline and follow-up samples were analyzed with each other by Wilcoxon matched pairs signed rank test. Correlations between HE4 levels and other clinical and laboratory parameters were determined using Spearman’s test. The area under the receiver operating characteristic curve (ROC-AUC) value was determined for baseline HE4 to indicate the severity of COVID-19 disease and the clinical outcome. The maximum of Youden index was determined to identify the cut-off values. After converting continuous values, except for gender, into binary forms based on their medians, binary logistic regression analysis was used for baseline HE4, and other dependent demographical (age and sex) and laboratory variables for the prediction of disease severity and outcome. Statistical significance was defined when *P* value was < 0.05.

## Results

### Baseline clinical features of COVID-19 patients and sepsis controls

In total, 99 COVID-19 individuals were involved in this study to analyze serum HE4 levels upon ICU or outpatient clinic admission and during hospital treatment for predicting disease severity and progression. These patients were divided into three sub-groups according to their symptoms and clinical characteristics (Table 1). Regarding age and sex, there was no difference among these COVID-19 subgroups. Additionally, the length of hospital stay was also similar between the two hospitalized COVID-19 cohorts (median [IQR], 9.5 [6-18^6^–^18^] vs. 10 [6-13^6^–^13^] days). Mechanical ventilation was more frequently applied among critically ill COVID-19 patients with ARDS vs. those with severe pneumonia (37 vs. 2 individuals, *P* < 0.0001). Consequently, the mortality ratio was significantly higher within the critically ill patient group (90% vs. 7.5%, *P* < 0.0001).

Based on the routine laboratory tests, general inflammatory markers, such as CRP, PCT, IL-6, ferritin, and white blood cell  (WBC) count were significantly higher (*P* < 0.001 or *P* < 0.0001, respectively) in the critically ill cohort compared to severe COVID-19 subjects, while PLT count did not show a significant difference between the two COVID-19 groups. In addition, there were significant differences between severe and mild COVID-19 patients, especially in CRP, ferritin and total LDH activity. We also recruited 25 age- and sex-matched non-COVID-19 bacterial sepsis subjects as controls for comparison to critically ill COVID-19 patients. Although the latter COVID-19 study group showed higher SOFA scores than sepsis controls (12 [10–14] vs. 9 [5–10], *P* < 0.0001), significantly higher PCT, WBC counts and MPV values with lower PLT counts as well as worse renal function were detected in critically ill sepsis COVID-19 patients. Overall, the mortality ratio was lower among these controls than COVID-19 non-survivors (Table 1).

To evaluate the clinical status of the lung, and altered oxygenation of the organs, Horowitz-index was calculated in case of all patients suffered from COVID-19. In the presence of need for mechanical ventilation in most COVID-19 patients, the Horowitz-index was significantly lower in the cohort of critically ill vs. severe COVID-19 patients (95 [65–139] vs. 154 [100–277], *P* < 0.001) (Table 1).

### Increased baseline serum HE4 correlates with COVID-19 disease severity

**Fig. 1:**
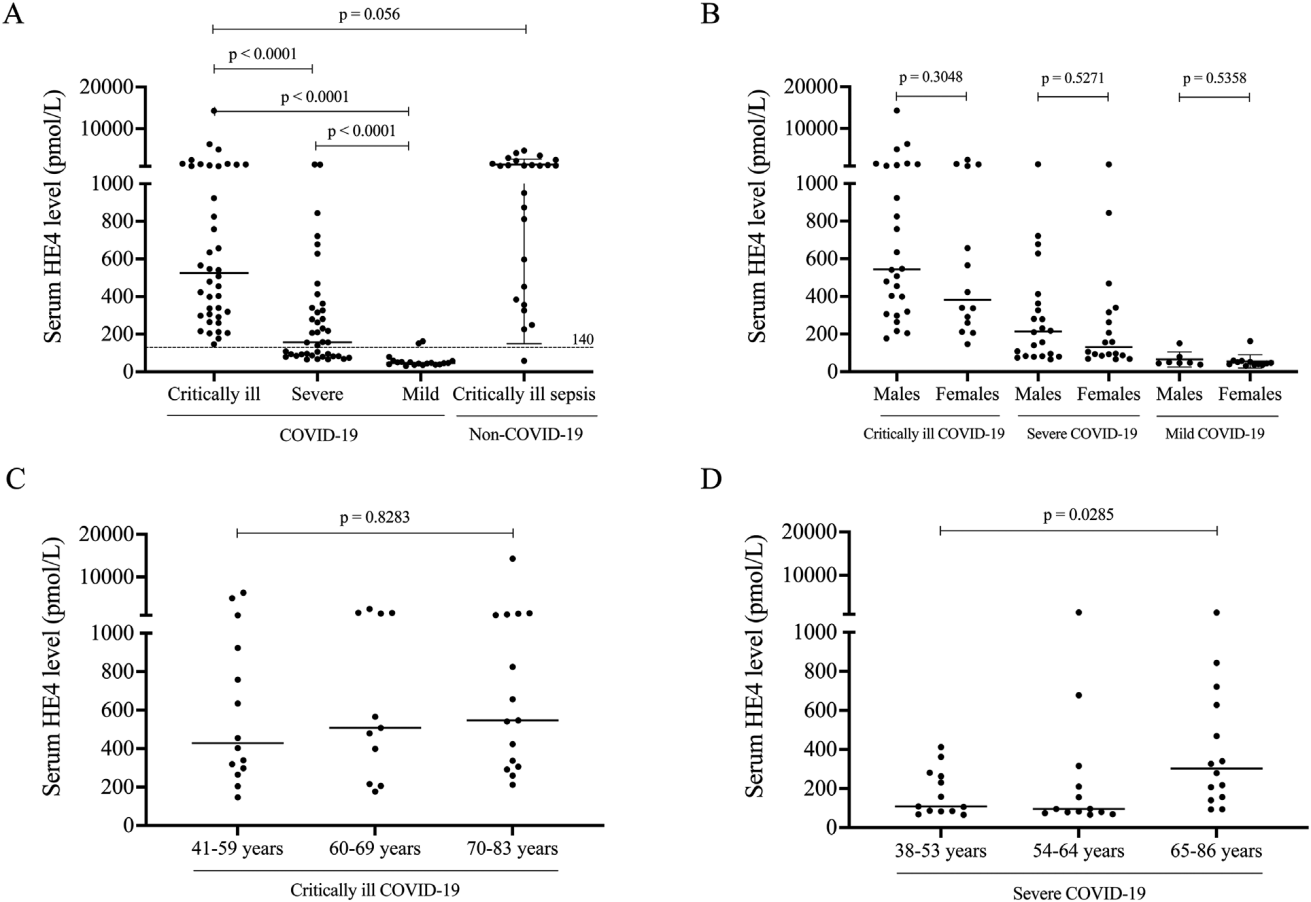
Comparison of baseline serum HE4 in COVID-19 patients based on disease severity in contrast to non-COVID-19 severe sepsis subjects. Significantly higher baseline level of HE4 was measured in critically ill (*n* = 40) vs. severe COVID-19 subjects (*n* = 40) as well as mild COVID-19 patients (*n* = 19), while similarly elevated values were found in subjects with non-COVID-19 sepsis (*n* = 25) than critically ill COVID-19 **(A)**. There were relatively higher HE4 levels in males than females in all subgroups, however, sex did not have a significant impact on HE4 **(B)**. Age did not significantly influence HE4 concentrations in critically ill **(C)** and mild COVID-19 patients (*P* = 0,8015) (data not shown), while there was an increasing tendency in HE4 in the severe COVID-19 cohort **(D)**. Dots represent single results, while bars indicate median value. In part A, the dashed line represents the cut-off value of HE4 (< 140 pmol/L) used in postmenopausal individuals. To compare the data of two groups, Mann-Whitney U test was applied

**Fig. 2:**
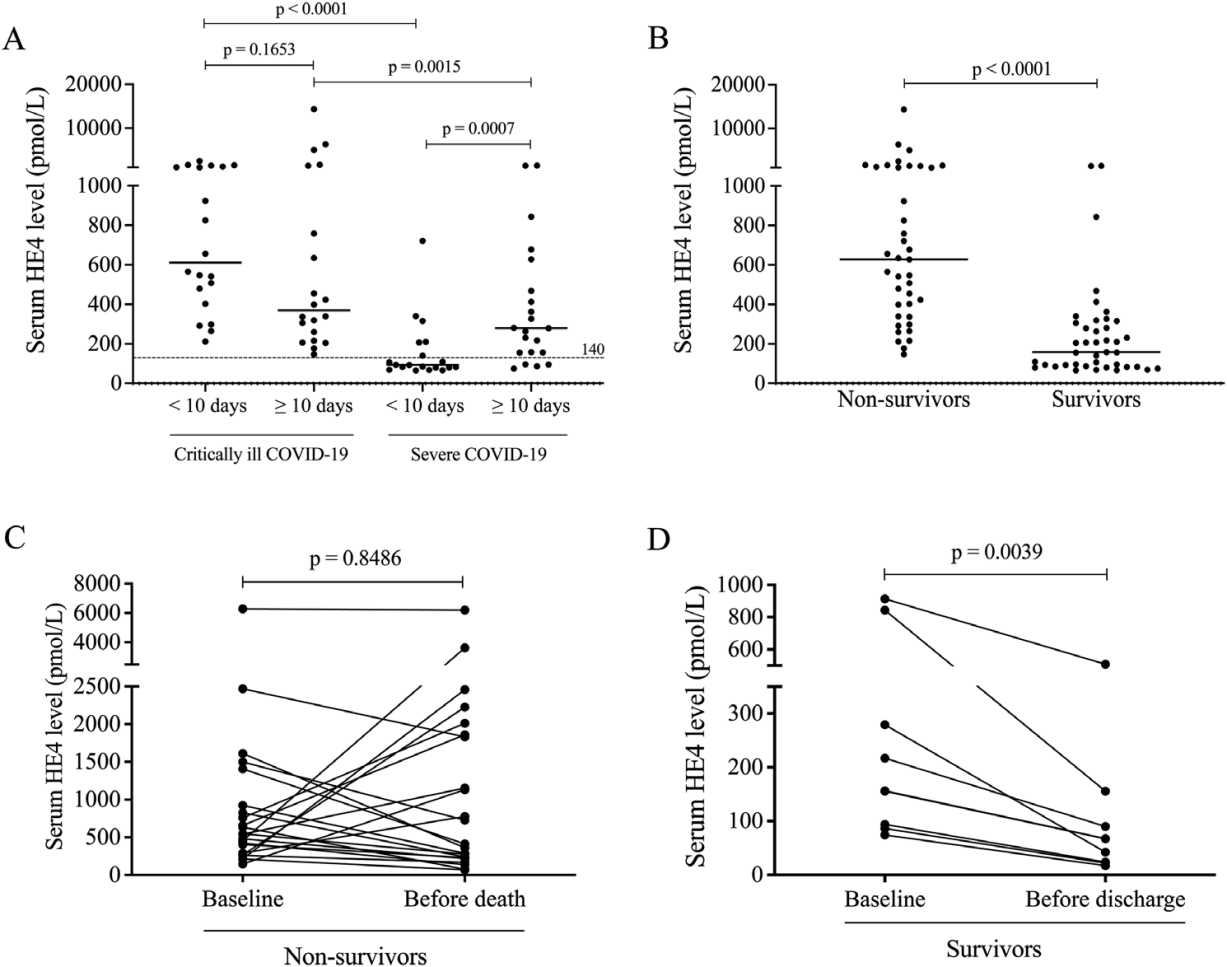
The association between baseline HE4 and the length of hospital stay **(A)** as well as the outcome of COVID-19 (**B–D**). There was no significant difference in HE4 in relation to shorter or longer hospitalization within the critically ill cohort, while those severe patients who had lower baseline HE4 level stayed for less days at hospital **(A)**. A significant difference was observed in serum HE4 between survivors (*n* = 41) and non-survivors (*n* = 39) **(B)**. When the alteration in HE4 level was studied during the follow-up regarding the outcome of these COVID-19 cases (*n* = 30), only a modest, non-significant change in HE4 was seen in those who died of COVID-19 eventually (**C**), while a significant decrease in HE4 between the baseline and follow-up sample was detected in those who recovered after treatment **(D)**. Dots represent single results, while bars indicate median value. In parts **C** and **D**, lines connect HE4 values measured in baseline and follow-up samples. To compare the data of two groups, Mann-Whitney U test was applied, while HE4 values in baseline and follow-up samples were analyzed with each other by Wilcoxon matched-pairs signed rank test

First, baseline serum HE4 level of each COVID-19 patient was retrospectively measured (Fig. 1A). Individuals under critically ill COVID-19 clinical conditions had a significantly higher baseline HE4 level than those in the severe or mild COVID-19 cohort (*P* < 0.0001). Importantly, all HE4 concentrations in the critically ill cohort were above the cut-off value of HE4 used in postmenopausal populations [20], while 45% of severe COVID-19 subjects showed normal HE4 levels based on this reference value. Due to mild SARS-CoV-2 infection, only 2 patients showed higher HE4 levels than 140 pmol/L. When HE4 results of the critically ill COVID-19 cohort were compared to the non-COVID-19 sepsis patients, similarly high levels were observed but there was no significant difference between the two groups (*P* = 0.056) (Fig. 1A). Next, the potential effect of age and sex was examined on HE4 serum levels in COVID-19. Sex-related differences were not found in either COVID-19 cohorts (Fig. 1B). When the relationship between age and HE4 was investigated, Spearman’s test showed a significant correlation between these variables (*r* = 0.349, *P* = 0.0015) (Table 2). When COVID-19 patients showing different disease severity were separately investigated, no alteration was seen among critically ill (*P* = 0.8283) (Fig. 1C) and mild COVID-19 participants (*P* = 0.8015) (data not shown), however, there was still an increasing tendency in HE4 levels among severe subjects by age (*P* = 0.0285) (Fig. 1D). We also examined the correlation between serum HE4 and the routinely determined laboratory parameters. Our data confirmed that baseline HE4 level demonstrated a moderate but statistically significant positive correlation with general inflammatory markers, such as CRP, IL-6, and WBC counts, certain prognostic markers of COVID-19, e.g., total LDH activity, ferritin, and serum ACE2 activity [19] and finally some important clinical parameters including the SOFA score and the degree of lung manifestation based on chest CT scanning (Table 2). In terms of the correlation between the length of hospital stay and HE4, we found an inverse trend between critically ill and severe patients. Under severe clinical conditions, significantly higher HE4 concentrations (*P* = 0.0007) were measured in those survivors who remained longer at hospital (≥ 10 days), while critically ill COVID-19 with higher HE4 levels (*P* = 0.1653) died earlier with shorter hospital stay (Fig. 2A). These results suggest that serum HE4 level before treatment was highly modulated by the severity of the developed COVID-19 clinical status in association with massive systemic inflammation and pulmonary dysfunction.

**Table 2:** Correlations between baseline HE4 level and some important clinical and routine laboratory parameters. Spearman’s test was performed when HE4 showed a moderate but statistically significant correlation with inflammation specific biomarkers, such as CRP, IL-6, ferritin, and WBC count. Among hematology parameters, HE4 had a positive association with serum ACE2 activity and PLT count, but inversely correlated with renal function. Abbreviations: CRP: C-reactive protein, IL-6: interleukin-6, AST: aspartate transaminase, ALT: alanine aminotransferase, LDH: lactate dehydrogenase, GFRCKD-EPI: Glomerular filtration rate - Chronic Kidney Disease Epidemiology Collaboration, ACE2: Angiotensin-converting enzyme 2, WBC: white blood cell, PLT: platelet

Demographical variables	Baseline HE4 (pmol/L)
Age (years)	*r* = 0.349, *p* = 0.0015
SOFA-score	*r* = 0.659, *p* < 0.0001
Lung manifestation based on CT (%)	*r* = 0.353, *p* = 0.0096
**Laboratory parameters**	
CRP (mg/L)	*r* = 0.407, *p* = 0.0002
IL-6 (ng/L)	*r* = 0.534, *p* < 0.0001
Ferritin (µg/L)	*r* = 0.502, *p* < 0.0001
Total LDH activity (U/L)	*r* = 0.431, *p* < 0.0001
GFR CKD-EPI (mL/min/1.73 m^2^)	r = -0.479, *p* < 0.0001
ACE2 activity (mU/L)	*r* = 0.434, *p* < 0.0001
WBC count (G/L)	*r* = 0.312, *p* = 0.0049
PLT count (G/L)	*r* = 0.231, *p* = 0.0395

### Kinetics of serum HE4 level in COVID-19 survivors and non-survivors

Recruited COVID-19 subjects were also divided into two other sub-cohorts according to the outcome of the disease, and baseline HE4 values were retrospectively investigated whether this biomarker could imply the outcome of COVID-19 before ICU treatment. Non-survivors had significantly higher HE4 levels (*P* < 0.0001) at hospital admission than survivors (Fig. 2B). Serum samples were available in case of 30 COVID-19 patients (22 non-survivors and 8 survivors) to monitor HE4 levels during hospital stay. In response to ICU medication, HE4 concentration tended to decrease, but no significant overall alteration was observed in non-survivors (*P* = 0.8486). In contrast, there was a significant reduction in serum HE4 before discharge compared to baseline values among those patients who recovered under treatment (*P* = 0.0039) (Fig. 2C and D). These results underline that baseline HE4 predicted the outcome of severe COVID-19, and its change during hospitalization also successfully followed the disease progression.

### Effectiveness of baseline serum HE4 level to indicate disease outcome and severity of COVID-19

**Fig. 3:**
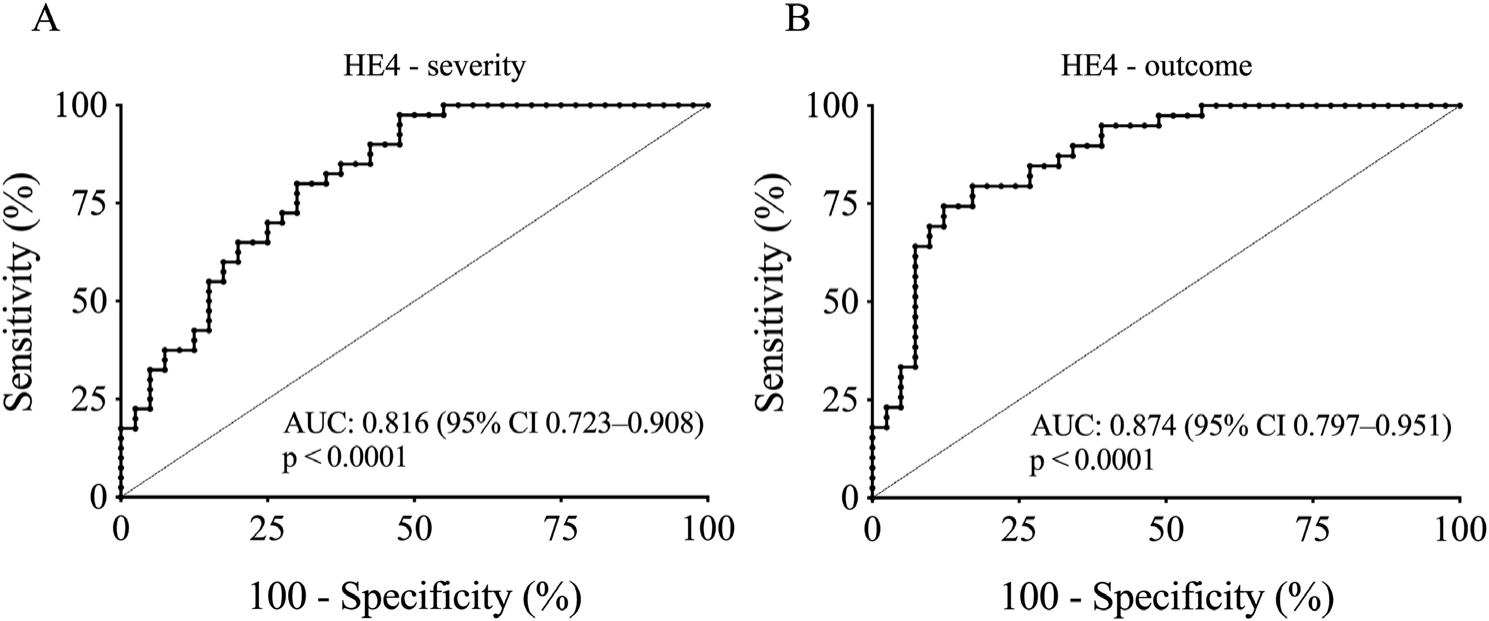
ROC-curve analysis for baseline HE4 levels for the prediction of the severity **(A)** and outcome of COVID-19 disease (**B**). The best discriminative cut-off value of serum HE4 at admission was 286.3 pmol/L with a sensitivity of 80% and specificity of 70% to estimate disease severity **(A)**. Using the cut-off value of 331.7 pmol/L, pre-treatment HE4 level could predict the outcome of the disease with a sensitivity of 80% and specificity of 83% **(B)**. ROC-AUC values with *P*-values were determined during these calculations

To further evaluate the clinical role of serum HE4 as a new prognostic inflammatory biomarker in COVID-19, we statistically analyzed its diagnostic characteristics to predict the severity and the outcome of this disease. For this purpose, ROC-AUC curve analyses were performed. The best discriminative threshold of HE4 level at admission, estimated by the Youden-index, was 286.3 pmol/L with a sensitivity of 80% and a specificity of 70% to estimate disease severity at an AUC value of 0.816 (95% CI [0.723–0.908], *P* < 0.0001). Moreover, in terms of mortality, the ideal cut-off value of baseline HE4 was 331.7 pmol/L with a sensitivity of 80% and a specificity of 83% to predict the outcome of COVID-19 with an AUC value of 0.874 (95% CI [0.797–0.951], *P* < 0.0001) (Fig. 3A and B). Based on these results, baseline HE4 was effective to assess the progression of life-threatening COVID-19 disease.

Binary logistic regression analysis was also performed to test whether serum HE4 could independently indicate either the disease severity or the clinical outcome considering other laboratory parameters of these COVID-19 individuals (Table 3). We found that elevated initial serum HE4 level showed a significantly high adjusted odds ratio (OR) for death (OR: 10.618 [2.331–48.354]; *P* = 0.002). In addition, reduced absolute lymphocyte count (OR: 0.175, 95% CI [0.033–0.925], *P* = 0.040), higher IL-6 (OR: 5.605 [1.516–20.724]; *P* = 0.010), elevated ferritin (OR: 5.911 [1.194–29.259]; *P* = 0.029) and increased WBC count (OR: 5.435 [1.152–25.626]; *P* = 0.032) also had a significant OR value in predicting clinical outcome. To predict the severity of the disease, baseline HE4 level demonstrated a “borderline” significant OR value (OR: 3.642, 95% CI [0.985–13.457], *P* = 0.053), while ferritin (OR: 4.813 [1.237–18.723]; *P* = 0.023) and absolute lymphocyte count (OR: 0.224 [0.055–0.909]; *P* = 0.036) showed a statistically significant OR (Table 3). Overall, these results support the usefulness of serum HE4 level as a potential prognostic biomarker in case of COVID-19.

**Table 3:** Binary logistic regression analysis for testing baseline HE4 levels to predict individually the outcome and severity of COVID-19. Key demographical and some laboratory parameters were considered as confounders for this calculation. We have categorized all continuous variables, except for gender, into binary forms based on their medians (in case of HE4: 286.3 and 331.7 pmol/L, respectively). Abbreviations: CRP: C-reactive protein, IL-6: interleukin-6, ACE2: Angiotensin-converting enzyme 2, WBC count: white blood cell count, Lym count: lymphocyte count, PLT count: platelet count

**Death/survival**	**Adjusted OR**	**95% CI**		***P*** **-value**
Age_bin	1.612	0.401	6.476	0.501
Gender	0.631	0.154	2.584	0.523
**HE4_bin**	**10.618**	**2.331**	**48.354**	**0.002**
CRP_bin	0.466	0.083	2.607	0.385
ACE2_bin	0.515	0.108	2.443	0.404
Ferritin_bin	5.911	1.194	29.259	0.029
Lym count_bin	0.175	0.033	0.925	0.040
PLT count_bin	1.155	0.257	5.181	0.850
IL-6_bin	5.605	1.516	20.724	0.010
WBC count_bin	5.435	1.152	25.626	0.032
**Critical/severe**	**Adjusted OR**	**95% CI**		***P*** **-value**
Age_bin	1.117	0.322	3.868	0.861
Gender	0.995	0.290	3.413	0.994
**HE4_bin**	**3.642**	**0.985**	**13.457**	**0.053**
CRP_bin	0.846	0.200	3.568	0.820
ACE2_bin	1.027	0.285	3.702	0.967
Ferritin_bin	4.813	1.237	18.723	0.023
Lym count_bin	0.224	0.055	0.909	0.036
PLT count_bin	0.971	0.253	3.728	0.967
IL-6_bin	3.131	0.979	10.010	0.054
WBC count_bin	3.281	0.859	12.530	0.082

### Prediction of 30-day mortality by augmented baseline HE4 level in patients with COVID-19

**Fig. 4:**
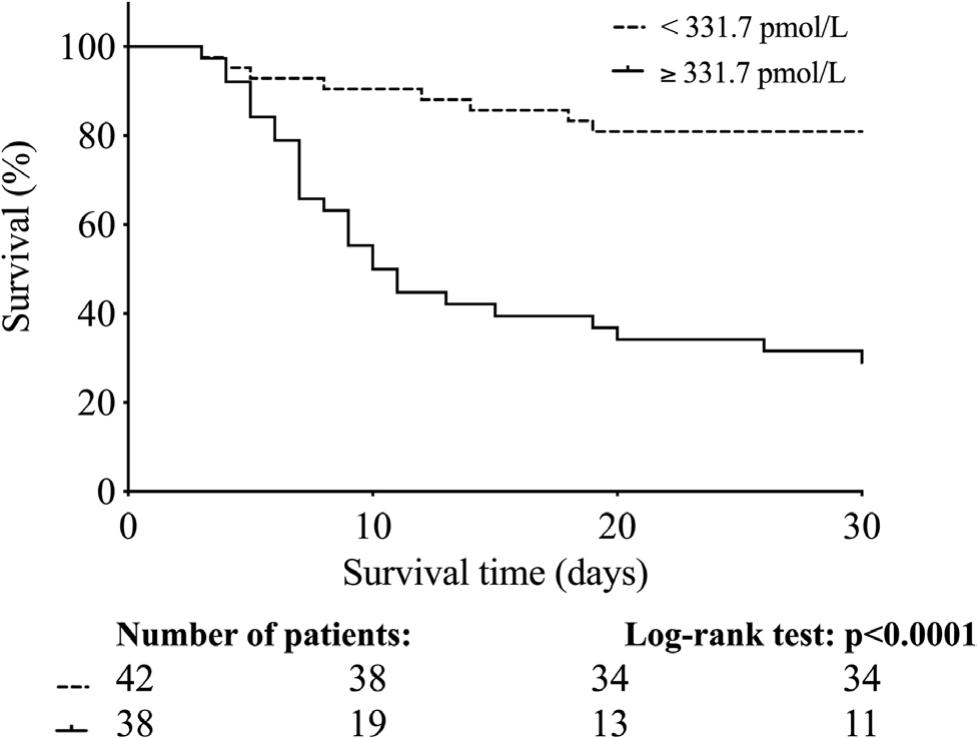
Kaplan-Meier analysis indicates that highly elevated HE4 levels were related to a higher rate of 30-day mortality. Cut-off value (≥ 331.7 pmol/L) was determined by the ROC-curve analysis. There was a lower risk of death for those COVID-19 subjects who had less than 331.7 pmol/L of HE4 level before treatment. Number of patients at risk are displayed at given days. Log rank *P* value was determined

Out of the 80 recruited ICU COVID-19 patients, 35 died during the 30-day follow-up. As we stated above, there was a significant difference in baseline HE4 serum level between COVID-19 survivors and non-survivors, and based on the ROC-AUC curve analysis, this biomarker efficiently estimated the outcome of this disease at the cut-off value of 331.7 pmol/L (see Fig. 3B). Using this cut-off value in the Kaplan-Meier analysis, COVID-19 patients with highly elevated HE4 levels had a significantly higher risk for 30-day mortality compared to those with lower HE4 concentrations (with a death ratio of 71% vs. 19%, respectively, Log rank *P* < 0.0001) (Fig. 4). When we further followed the clinical status of these patients up to 40 days, 4 more subjects died of COVID-19 in the critically ill population, while no other death case was recorded among those with < 331.7 pmol/L (data not shown). Taken together, serum HE4 level at ICU admission possessed a capacity to predict the outcome of COVID-19.

## Discussion

Anti-protease HE4 is a small secretory glycoprotein with a functional role in host defense of the lung [9]. In different cancers, such as ovarian and lung tumors, HE4 level can be highly expressed, which makes it a reliable follow-up tumor marker under these malignant conditions [10, 11]. On the other hand, recent clinical studies revealed its significance as a novel pulmonary inflammatory laboratory parameter in monitoring of treatment in cystic fibrosis patients [21–23], and as a diagnostic marker in sepsis-related ARDS [13, 14], COVID-19 disease [15, 16], and lung fibrosis [24]. The two main reasons why the analysis of HE4 came to focus as a relevant biomarker in severe SARS-CoV-2 infection were that (i) ARDS has a fibroproliferative phase in its subacute stage when profibrotic mediators are produced at a higher quantity [25], and (ii) in SARS-CoV-2 ARDS, there is a protease-anti-protease imbalance in the airways causing elevated expression of the major serine protease inhibitor alpha1-antitypsin induced by IL-6 [26]. Although the role of HE4 is not fully understood in COVID-19 [15, 16], we expected to measure highly increased HE4 concentrations due to the development of massive intrapulmonary inflammation [12, 25, 26], especially in the sera of patients suffering from critically ill COVID-19 but without cancer.

Hence, we conducted a clinical study to further characterize the alterations of serum HE4 level in critically ill, severe, and mild COVID-19 cohorts having comparable demographical parameters and applied a bacterial sepsis group as ICU controls to match their laboratory parameters (Table 1). Our critically ill COVID-19 patients demonstrated an extremely high ratio of severe lung manifestation with a median of 70% involvement of the lung (IQR: 55–80%), and a huge demand of mechanical ventilation (93% of these patients). Furthermore, regarding the widely used „Berlin clinical definition” of the diagnosis of ARDS [17], our recruited individuals fully met the criteria for critically ill clinical status. We measured significantly higher baseline HE4 levels among critically ill COVID-19 subjects compared to those within the severe COVID-19 group which were much higher vs a general postmenopausal population (< 140 pmol/L) [20]. Similarly high HE4 concentrations were reported by others in either non-COVID-19 ARDS [13, 14] and in COVID-19 disease [15, 16]. These data suggest that serum HE4 cannot discriminate COVID-19 and non-COVID-19 severe pulmonary conditions from each other, however, it shows a large difference in distinct severity of COVID-19 lung disease. Higher serum ACE2 activity - implicated in the pathomechanism of various cardiovascular diseases and a risk for COVID-19 mortality - was also determined in the critically ill vs severe COVID-19 which was identical with our recent findings [19]. Age affected HE4 concentrations only in those with severe pneumonia as described in sepsis-related ARDS [14], while sex had an influence on HE4 level in neither our current study nor previous COVID-19 investigations [15, 16]. No former data was available in terms of the correlation between the length of hospital stay and HE4 in COVID-19. Here we found an inverse trend between critically ill and severe patients when significantly higher HE4 concentrations were measured in those survivors who remained longer at hospital, while critically ill COVID-19 with higher HE4 levels died earlier with shorter hospital stay which correlates with the findings of a recent bacterial sepsis study [13].

Next, baseline serum HE4 was analyzed for the first time whether it had a capacity to predict the outcome of the disease. Non-survivors who were mostly the members of the “critically ill” sub-cohort had significantly higher HE4 values compared to survivors, similar to deceased cases with bacterial sepsis [14]. In former COVID-19 studies, serum HE4 was measured in only one SARS-CoV-2 positive sample of participants [15, 16]. Here, the kinetics of HE4 was parallelly studied during the hospitalization matching the baseline and the follow-up HE4 results. In case of non-survivors, we did not find a significant change in HE4 level, while among survivors there was a significant decrease in the quantity of HE4 due to treatment efficacy. In agreement with our data, significantly lowered post-treatment HE4 concentrations were reported in critically ill sepsis patients under successful ICU therapy [13]. We also note that in the critically ill cohort, continuous renal replacement therapy was also applied in 54% of patients due to acute kidney dysfunction that might affect the level of HE4 based on our previous data [20]. Importantly, no significant difference (*P* = 0.1521) was found among those critically ill subjects with and without CRRT in terms of delta HE4 (data not shown). To the best of our knowledge, there was no drug or other therapeutic procedure that substantially influenced the kinetics of serum HE4 levels in these patients.

On the hand, as we mentioned above, a recent investigation found elevated serum HE4 in idiopathic pulmonary fibrosis, which can be characterized by a recurrent alveolar epithelial cell damage and a dysregulated epithelial repair [24]. Presumably, after the acute phase of ARDS in COVID-19 a normal proliferative phase begins with effective reparation [12], which leads to lung recovery in patients showing decreasing HE4 levels and favorable outcome. Overall, HE4 not merely correlates with the disease severity, but also reflects the progression of COVID-19.

Serum HE4 upon admission and routinely determined laboratory parameters were correlated with each other using Spearman’s test. A moderate but statistically significant positive correlation with general inflammatory markers, such as CRP, IL-6, WBC count, total LDH activity, ferritin, and serum ACE2 activity. These results agree with previous data in COVID-19 [15, 16] or sepsis ARDS [13, 14]. Moreover, some important clinical parameters including the SOFA score and the degree of lung manifestation based on chest CT scanning were largely correlated with baseline HE4 (Table 2). Likewise, sepsis-related ARDS patients had a substantial positive correlation between HE4 and the SOFA as well as APACHE II score [13, 14].

We then examined the suitability of HE4 serum level to predict the severity and the outcome of COVID-19 using ROC-AUC curve analyses. To estimate the severity of the disease, the best discriminative threshold of HE4 serum level at the hospital admission was 286.3 pmol/L at an AUC value of 0.816 (Fig. 3A). Even higher AUC values (0.920) were reported in another COVID-19 population at a cut-off value of 359 pmol/L [15]. To predict the COVID-19 associated mortality, the ideal cut-off value of the HE4 quantity was as high as 331.7 pmol/L at a substantial AUC value of 0.874 in our study (Fig. 3B). In sepsis-ARDS, serum HE4 recently showed a comparable AUC value of 0.881 for predicting 28-day mortality [13]. To confirm these relationships, binary logistic regression analysis was also performed to test whether serum HE4 could independently indicate either the disease severity or the clinical outcome. We found that elevated initial serum HE4 level showed a high OR value for unfavorable outcome (OR: 10.618) and more severe disease (OR: 3.642). Our OR values were much higher than what others reported (OR: 1.007), however, those authors used a multivariate analysis for the prediction of ARDS in sepsis [14]. Interestingly, ACE2 activity in this COVID-19 cohort did not show a significant OD value in either aspect in contrast to our recent publication [19].

Finally, we analyzed the cut-off value of baseline HE4 to estimate the outcome of COVID-19 by Kaplan-Meier curve analysis. COVID-19 patients with highly elevated baseline HE4 levels (≥ 331.7 pmol/L) had a significantly higher risk for 30-day mortality compared to those with somewhat lower HE4 concentrations (Log rank *P* < 0.0001). Survival curves were earlier analyzed in septic subjects with ARDS, and 1361 pmol/L cut-off value was set for a higher risk of death at 28 days at ICU [13]. Overall, these data underline that serum HE4 measurement at ICU admission is useful to predict COVID-19 outcome.

This two-center study within one university hospital has some limitations. The sample size is limited, however, it was acceptable based the sample size calculation, and it is comparable to the two previous studies published on serum HE4 in COVID-19 patients [15, 16] and other sepsis-related ARDS subjects using the same analytical platform. Furthermore, this is the only study so far measuring this parameter in terms of clinical outcome of COVID-19. Despite these limitations, our study provides highly anticipated data regarding the efficiency of HE4 analysis in critically ill COVID-19 population. However, further studies are required to support these clinical data and to discover its pathophysiological function in the mechanism of SARS-CoV-2 induced severe acute lung injury.

## Conclusions

Serum HE4 may be an early-stage biomarker for stratification of life-threatening COVID-19 status that highly correlates with lung disease severity and predicts clinical outcome.

## Data Availability

The datasets generated and/or analyzed during the current study are not publicly available but are available from the corresponding author on reasonable request.
